# The pro-angiogenesis effect of miR33a-5p/Ets-1/DKK1 signaling in ox-LDL induced HUVECs

**DOI:** 10.7150/ijbs.60302

**Published:** 2021-10-03

**Authors:** Mingxue Di, Yu Zhang, Renya Zeng, Xiaolin Liu, Weijia Chen, Meng Zhang, Cheng Zhang, Mengmeng Li, Mei Zhang

**Affiliations:** 1 The Key Laboratory of Cardiovascular Remodeling and Function Research, Chinese Ministry of Education, Chinese National Health Commission and Chinese Academy of Medical Sciences, The State and Shandong Province Joint Key Laboratory of Translational Cardiovascular Medicine, Department of Cardiology, Qilu Hospital of Shandong University, Jinan, China.; 2 Department of Gerontology, The First Affiliated Hospital of Shandong First Medical University & Shandong Provincial Qianfoshan Hospital.

**Keywords:** DKK1, Ets-1, miR33a-5p, angiogenesis, HUVECs

## Abstract

**Objective:** Angiogenesis is involved in multiple biological processes, including atherosclerosis (AS) and cancer. Dickkopf1 (DKK1) plays many roles in both tumors and AS and has emerged as a potential biomarker of cancer progression and prognosis. Targeting DKK1 is a good choice for oncological treatments. Many anticancer therapies are associated with specific cardiovascular toxicity. However, the effects of DKK1 neutralizing therapy on AS are unclear. We focused on how DKK1 affected angiogenesis in AS and ox-LDL-induced human umbilical vein endothelial cells (HUVECs).

**Methods:** ApoE-/- mice were fed a high-fat diet and then injected with DKK1i or DKK1 lentivirus to study the effects of DKK1. *In vitro*, promoter assays, protein analysis, database mining, dual-luciferase reporter assay (DLR), electrophoretic mobility shift assay (EMSA), chromatin immunoprecipitation (ChIP), and coimmunoprecipitation (co-IP) were used to study the mechanism of DKK1 biogenesis. Cell migration and angiogenesis assays were performed to investigate the function and regulatory mechanisms of DKK1.

**Results:** DKK1 participated in angiogenesis both in the plaques of ApoE-/- mice by knockdown or overexpression of DKK1 and ox-LDL-induced HUVECs. DKK1 induced angiogenesis (increasing migration and capillary formation, inducing expression of VEGFR-2/VEGF-A/MMP) via the CKAP4/PI3K pathway, independent of Wnt/β-catenin. ox-LDL increased the expression and nuclear transfer of Ets-1 and c-jun, and induced the transcriptional activity of DKK1 in HUVECs. Ets-1, along with c-jun and CBP, could bind to the promoter of DKK1 and enhance DKK1 transcription. MiR33a-5p was downregulated in ox-LDL induced HUVECs and aortic artery of high-fat diet ApoE-/- mice. Ets-1 was a direct target of miR33a-5p. MiR33a-5p/Ets-1/ DKK1 axis contributed to angiogenesis.

**Conclusions:** MiR33a-5p/Ets-1/DKK1 signaling participated in ox-LDL-induced angiogenesis of HUVECs via the CKAP4/PI3K pathway. These new findings provide a rationale and notable method for tumor therapy and cardiovascular protection.

## Introduction

Dickkopf-1 (DKK1), a secreted inhibitor of the canonical Wnt/β-catenin pathway, plays complex cellular and biological roles in different diseases. DKK1 is overexpressed in bone pathologies and many cancers, has now emerged as a potential biomarker of cancer progression and prognosis for several types of malignancies 1, and has been shown to have immunosuppressive effects 2. DKK1 has been widely investigated in oncology and is now considered a promising target for anticancer therapy 1. For example, DKN-01 is an IgG4 clinical stage antibody that potently and specifically neutralizes human and murine DKK1 and was used in a recently completed promising study in combination with pembrolizumab in patients with gastric/gastroesophageal junction cancer 3. The treatment outcomes for a wide range of malignancies have improved remarkably due to the development of many novel anticancer therapies, including vascular endothelial growth factor inhibitors (VEGFIs). However, as a side effect, oncological treatment may increase the morbidity and mortality of cardiovascular diseases (CVDs), including via acceleration of atherosclerosis (AS) 4. We confirmed that DKK1 induced endothelial cell (EC) dysfunction and AS 5. Other data also suggest that DKK1 is an important driver of the initiation and progression of AS and a promising target for atheroprotection 6. DKN-01 was evaluated in a phase I multicenter study for advanced tumor therapy, and better outcomes were associated with biomarkers of angiogenesis inhibition, which indicated the potential antiangiogenic and immunomodulatory activity of DKN-01 7. Because there exist cross-susceptibility factors and common targets between tumors and CVDs, elucidating the regulatory effect and molecular mechanism of DKK1 in EC angiogenesis will provide a theoretical basis and clinical reference for the identification of new effective intervention targets for antitumor drugs.

ECs, located on the surface of the vascular wall, are always vulnerable to various risk factors, such as hypertension and hyperlipidemia. In our previous study, we found that DKK1 induces endothelial dysfunction in plaques and human umbilical vein endothelial cells (HUVECs) 8. Angiogenesis, which provides essential oxygen and nutrients for proliferation and metastasis, is an indispensable process for tumor growth and metastatic dissemination. Tumor angiogenesis has become a new and promising target for antitumor therapy. While angiogenesis also plays an important role in AS, we focused on the effect of DKK1 on angiogenesis.

DKK1 has two cysteine rich domains (CRDs): CRD-N and CRD-C. DKK1 binds to LRP6 and antagonizes the downstream canonical Wnt pathway. In addition, the CRD-N of DKK1 binds to cytoskeleton-associated protein 4 (CKAP4), and then, the intracellular segment recruits PI3K and activates AKT. DKK1 induces angiogenesis through Wnt/β-catenin-dependent or Wnt/β-catenin-independent mechanisms in tumor cells. However, the downstream mechanisms by which DKK1 induces angiogenesis in ox-LDL-induced HUVECs are unknown.

Studies have found several mechanisms upstream of DKK1, including histone modification, transcriptional changes, posttranscriptional changes and posttranslational modifications (phosphorylation, glycosylation) 9-12. However, the upstream mechanism underlying the upregulation of DKK1 in ox-LDL-induced HUVECs is still unknown. Promoter analysis by genome bioinformatics methods (such as JASPAR and ALGGEN) revealed that the DKK1 promoter can bind via multiple possible sites to the transcription factors Ets-1, c-jun, c-fos and so on. Under ox-LDL stimulation, we found that Ets-1 was obviously upregulated compared with other predicted transcription factors. Moreover, Ets-1 siRNA severely inhibited the upregulation of ox-LDL induced DKK1. Ets-1, a member of the E26 transformation-specific (Ets) family, possesses a conserved domain (EBS) that recognizes GGAA/T 13. Previous studies found that Ets-1 performed multiple functions in ECs: (1) Ets-1 directly regulated several vascular genes, such as Flt1, Tek, Kdr, Angpt2, Nrp1, vWF, PECAM1 and Cdh5, to promote angiogenesis 14; (2) Ets-1 directly upregulated MMPs (MMP1, MMP3, MMP9 and MMP13) and β3 integrin to promote migration in the nucleus 15, 16. However, it is unclear whether Ets-1 affects the function of ECs by regulating DKK1.

MicroRNAs are a class of noncoding single-stranded small RNA molecules that can specifically pair with the 3' untranslated region (3'-UTR) of target gene mRNAs to inhibit the expression of target genes through translational repression or mRNA degradation 17. One miRNA can target one or more genes, and the regulatory mechanism of a miRNA may be different in different cells. MiRNAs are regulators of vascular endothelial functions and AS. With the aid of well-known programs (such as miRBase, STARBASE and TargetScan), we found that miR33a-5p was markedly downregulated under ox-LDL stimulation in HUVECs. Both DKK1 and Ets-1 are target genes of miR33a-5p. However, whether miR33a-5p can bind to the 3'-UTR of DKK1 and Ets-1 mRNA to regulate their translation is unknown. Studies have found that miR33a-5p may be related to macrophage lipid metabolism 18 and inhibition of tumor cell proliferation 19. A previous study also found that DKK1 is regulated by miR33a in diabetic cardiomyopathy 20. However, the mechanisms of action of miR33a-5p in ECs and the function of miR33a-5p in regulating Ets-1 and DKK1 are not yet clear.

Based on these findings, we hypothesized that miR33a-5p/Ets-1 participates in the regulation of ox-LDL-induced DKK1 expression in HUVECs. To test this idea, we investigated the underlying upstream mechanisms of DKK1 expression in HUVECs angiogenesis. Identifying these pathways will improve our understanding of the regulation of tumor angiogenesis and provide new methods to address cardiovascular toxicity in antitumor therapy. An in-depth study of the effect of DKK1 on angiogenesis will provide a solid theoretical basis for improving the development of drugs to treat tumors and reduce or even protect against AS. It is hoped that the relevant new drugs will play a better synergistic role in the treatment of tumors and AS in clinical application.

## Materials and Methods

Please see the Major Resources Table in the Supplemental Materials.

### Ethics statement

All *in vivo* protocols involving animal care and experiments complied with the Guide for Care and Use of Laboratory Animals published by the United States National Institutes of Health (NIH Publication, 8th Edition, 2011) and the Animal Management Rules of the Chinese Ministry of Health (Document No. 55, 2001). All *in vivo* experiments were approved by the Animal Care Committee of Shandong University. All *in vitro* experimental protocols were approved by the Key Laboratory of Cardiovascular Remodeling and Function Research, Qilu Hosipital, China.

### Atherosclerosis animal model protocol and lentiviral gene transfer

A total of 120 ApoE-/- mice (eight- week- old males) were purchased from Beijing HFK Bioscience Co.,Ltd. All mice were fed atherogenic chow (i.e., a high-fat diet with 0.25% cholesterol and 15% cocoa butter) at 14 weeks. The atherosclerotic model was created as previously described. We applied constrictive silica collars to the right carotid artery (RCA) to accelerate atherosclerotic lesion formation at the third week. Pentobarbital sodium was used for anesthesia via intraperitoneal injection (40 mg/kg) when placing the constrictive collars. Eight weeks after the surgery, the mice were randomly divided into four groups (n=15 each): a normal saline group (NS), an empty lentivirus group (GFP), a DKK1i lentivirus group (shDKK1), and a DKK1 lentivirus group (DKK1). A 200 µl suspension (4×10^8^ TU DKK1i or DKK1 lentivirus per ml) was injected into each mouse through the tail vein. The mice were sacrificed 4 weeks posttransfection using pentobarbital sodium (50 mg/kg, i.p.) before exsanguination by perfusion via the abdominal aorta with PBS.

### Histopathology and immunohistochemistry

The RCAs were dissected, removed, fixed in 4% formaldehyde overnight at 4 °C, and embedded in OCT compound, and 5-μm-thick sections were prepared. After blocking in 5% bovine serum albumin (BSA) in PBS, the cryosections were incubated with primary antibodies overnight at 4°C and then with an HRP Detection System (ZSGB-BIO). Detection was subsequently performed using 3, 3′-diaminobenzidine (DAB) (ZSGB-BIO). Plaques stained with picrosirius red were viewed under polarized light. Staining in the plaque was quantified using Image-Pro Plus 6.0 software (Media Cybernetics, USA) and a color CCD video microscope (Olympus, Japan).

### Cell culture

HUVECs were obtained from ScienCell Research Laboratories (Carlsbad, CA, USA) and cultivated in endothelial cell medium (ECM) (ScienCell, Carlsbad, CA). HEK293T cells were obtained from the American Type Culture Collection (ATCC) and cultured in high-glucose Dulbecco's modified Eagle's medium (DMEM). The medium contained 10% fetal bovine serum (FBS) and 1% penicillin/streptomycin. Both cell lines were incubated in a humidified 5% CO_2_ incubator at 37 °C.

### Plasmid construction

Constructs with the wild-type and mutated 3'-UTRs of the human DKK1 gene (pGL3-DKK1-3'-UTR-WT and pGL3-DKK1-3'-UTR-MUT) and the wild type and mutated 3'-UTRs of the human Ets-1 gene (pGL3-Ets-1-3'-UTR-WT and pGL3-Ets-1-3'-UTR-MUT) were generated. The luciferase reporter gene plasmid (pGL3) and plasmids with the PGL3-basic-DKK1 promoter (P0), the PGL3-DKK1 promoter (P1-P9), and the PGL3-basic-DKK1 promoter (deletATGGAAT) (P0-del) were constructed, including the full-length DKK1 promoter, the serially truncated DKK1 promoter and deletion fragments of the DKK1 promoter, respectively. The PCDNA3.1-Ets-1 and PCDNA3.1-c-jun plasmids were constructed to overexpress Ets-1 and c-jun, respectively. All the plasmids were obtained from Shanghai GenePharma Biotechnology Company.

### siRNA and RNA interference

Upon reaching 40%-60% confluence, cells were transfected with specific siRNA or plasmids (GenePharma, Shanghai) using Lipofectamine 3000 (Thermo Fisher Scientific, USA) in Opti-MEM (Gibco, Thermo Fisher Scientific, USA). At 6 h after transfection, the medium was replaced. Cells were collected for detection of the luciferase reporter or of protein expression for 24-48 h after transfection.

### Immunofluorescence staining and microscopy

The cells were washed with PBS, fixed with 4% paraformaldehyde, penetrated with 0.5% Triton X-100 PBS, and incubated with primary antibodies at 4 °C overnight. The sections were washed with PBS, and incubated with FITC- conjugated secondary antibodies. Nuclei were stained with 4′, 6-diamidino-2-phenylindole (DAPI; 1:2000, Roche, Germany) for 5 min. The samples were rinsed three times in PBS and examined under an epifluorescence microscope, and the data were analyzed using Image-Pro Plus 6.0 software (Media Cybernetics, USA).

### Western blot analysis

HUVECs were lysed using RIPA buffer containing 1 mM PMSF and collected by centrifugation at 14,000 × rpm for 10 min. Proteins were separated on 10% SDS-PAGE gels, transferred to PVDF membranes with a 0.45 µm pore size (Millipore, USA), and incubated with primary antibodies overnight at 4 °C. The membranes were incubated with secondary antibodies the next day for 80 min. Bands were visualized using Immobilon ECL substrate (Millipore, USA), and imaged with an LAS-4000 luminescent image analyzer (Fujifilm, USA). Protein expression was quantified using Adobe Photoshop CS6 (Adobe Systems, USA) and normalized to β-actin expression in each sample; the expression level is shown as a percentage of the control.

### RNA extraction and quantitative real-time PCR

Total RNA was extracted from HUVECs using TRIzol reagent (Ambion, Life Technologies, USA), and reverse-transcribed into cDNA using a PrimeScript™ RT Reagent Kit (TakaRa Biotechnology, Dalian, China). cDNA (1 ng) was subjected to q-PCR using SYBR Green (TakaRa Biotechnology, Dalian, China) for the relative quantification of mRNA expression. Quantification was accomplished using the 2-ΔΔCt method. β-Actin was used to normalize mRNA levels. U6 was used to normalize microRNA levels.

### Dual-luciferase reporter assay

Cells were seeded in 24-well plates and cotransfected with 100 ng of reporter plasmid and 20 ng of pRL-TK by using Lipofectamine 3000 (Thermo Fisher Scientific, USA) in Opti-MEM (Gibco, Thermo Fisher Scientific, USA). After 48 h, cells (HUVECs or 293T cells) were harvested for the dual-luciferase assay. A dual-luciferase reporter (DLR) assay system (Genecopeia, USA) was used to measure the luciferase activity. The firefly luciferase values were normalized to Renilla luciferase activity before statistical analyses.

### Electrophoretic mobility shift assay

HUVECs were treated with ox-LDL for 6 h. Nuclear extracts were obtained using a NE-PER nuclear protein extraction kit (Thermo Scientific, Rockford IL, USA) according to the manufacturer's instructions. Double-stranded oligonucleotides were obtained by annealing equal amounts (0.1 mg) of the complementary single-stranded oligonucleotides by heating to 95°C for 5 min and then gradually cooling to room temperature. Then, 0.01 μmol of digoxigenin-labeled oligonucleotide probes was incubated with nuclear extracts in DNA binding buffer [10 mM Tris-HCl (pH 7.5), 1 mM MgCl_2_, 50 mM NaCl, 0.5 mM EDTA, 4% glycerol, and 0.5 mM 2,3-dihydroxy-l,4-dithiobutane (DTT)] and 1 µg of poly(dI-dC). A competition assay was performed using a 200-fold excess of cold probes or cold mutated probes (2 µmol), which were preincubated with the reaction mixture before the addition of biotin-labeled probes. To ascertain the specificity of the nuclear proteins bound to Ets-1 sites, a supershift assay was performed with 2 mg of Ets-1 antibody. After incubation for 30 min, DNA-protein complexes were separated by 6.0% nondenaturing PAGE (Invitrogen) and transferred to a nylon membrane. DNA was crosslinked by UV irradiation for 10 min. The nitrocellulose membrane was evaluated by the addition of a streptavidin-horseradish peroxidase conjugate and a chemiluminescent substrate. Then, the nitrocellulose membrane was imaged with an LAS-4000 luminescent image analyzer (Fujifilm, USA). A prominent single supershifted band was observed when nuclear extracts were incubated with an anti-Ets-1 antibody.

### Chromatin immunoprecipitation

Chromatin immunoprecipitation (ChIP) analysis was performed by a ChIP kit (CST, Boston, USA), according to the manufacturer's protocols. Cells (4×10^7^) were crosslinked with 4% formaldehyde, lysed, and enzymatically digested into 200-bp DNA fragments. The sheared chromatin was incubated with different antibodies and magnetic beads at 4°C overnight. Purified immunoprecipitated chromatin fragments from the IP samples were tested by PCR. The primers for the Ets-1 binding site in the human DKK1 promoter (-2080 to -1894) were as follows: 5′- ACACAGCTTGCAGATTTCCTAGT -3 ′ and 5 ′-TATGGTCTGTGTTCTAGTTCCTTCA -3′. qPCR was used for quantitative analysis of the ChIP enrichment efficiency and for expression analysis according to the 2^-△△CT^ method.

### Coimmunoprecipitation assay

Cells (4×10^7^) were lysed with 1 ml of RIPA for 30 min and centrifuged at 12000 × g and 4 °C for 30 min. Save 10 μl supernatant and added to 2× SDS loading buffer, denatured at 99 °C for 10 min standby. Pretreated protein A/G magnetic beads (Bimake, Shanghai, China) with 300 µl RIPA were added to 6 µg of antibody and then incubated 4 °C for 15 minutes. The mixture was placed on a magnet for 1 minute, removed the supernatant, and washed 3 times. The beads were resuspended by the sample (300 µl), then incubated at 4 °C overnight with gentle rotation. The mixture was placed on a magnet for 1 minute, removed the supernatant, and washed 3 times. The beads was added to 1× SDS loading buffer, denatured at 99 °C for 10 min, centrifuged 12000 × g for 10 min, saved supernatant and evaluated by Western blot.

### EdU cell proliferation assay

Five-ethynyl-2'-deoxyuridine (EdU) cell proliferation assays were carried out according to the manufacturer's instructions (RiboBio, Guangzhou, China) using the Cell-Light™ EdU imaging detection kit.

### Transwell assay

Transwell inserts are an array of 24 individual Boyden chambers with 8 µm pore size Transwell membranes (Corning, NY, USA). Cells were digested with trypsin and suspended in serum-free culture medium at 5×10^5^/well. Samples (200 µl) were placed in the upper chamber, and the lower chamber was filled with serum-free culture medium (500 µl). The cells were transfected and treated with ox-LDL at the designated concentrations and for the indicated times. The noninvading cells remained on the upper surface of the membrane and were removed with cotton swabs, whereas the cells that passed through the membrane were fixed with 4% formaldehyde, stained with 0.2% crystal violet, and then counted under an optical microscope after 24 h.

### Scratch wound assay

A thin mark was drawn vertically with a 20-µl pipette tip in the six-well plate. The cells were then washed three times with PBS to remove the floating and detached cells. Fresh serum-free medium was added. The migratory distance was measured 0 h, 6 h, 12 h, and 24 h after wounding using IPP software. Cell migration is expressed as the percentage of the open wound area at 24 h relative to that at 0 h.

### *In vitro* angiogenesis assay

Cells were transfected or treated with ox-LDL. A 12-well plate was precoated with Matrigel (BD Bioscience, Billerica, MA, USA). Cells were digested with trypsin, suspended in serum-free culture medium at 5×10^4^/well and seeded on the 12-well plates. The cells were stained by calcein-AM, and the angiogenic properties were assessed after 12 h. The tube length was measured using IPP software.

### Statistical analysis

The data were analyzed using SPSS v23.0 (SPSS Inc., Chicago, IL) and are presented as the mean ± SEM. of at least three independent experiments. Comparisons were analyzed using Student's t test or one-way ANOVA followed by the Bonferroni post hoc test. p<0.05 was considered statistically significant.

## Results

### DKK1 aggravated plaque-associated angiogenesis *in vivo*

**Figure 1:**
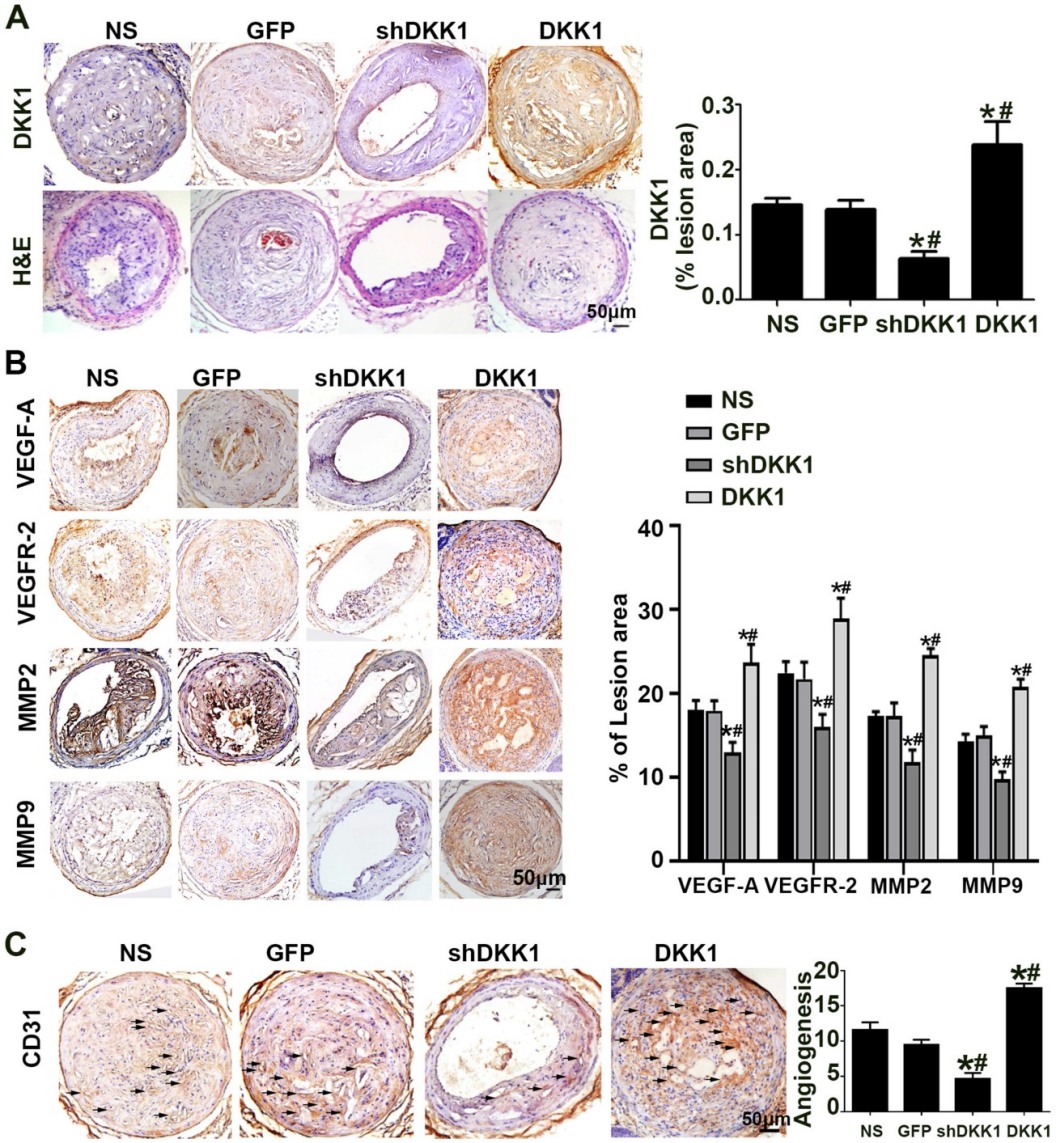
** Effect of DKK1 on angiogenesis in the plaques of ApoE-/- mice. (A)** Morphology of the RCAs, as shown by HE staining, and expression of DKK1 shown by DKK1. Bars indicate 50 µm. **(B)** Protein expression levels of VEGF-A, VEGFR-2, MMP-2 and MMP-9 in atherosclerotic plaques of ApoE-/- mice were determined by immunohistochemical staining. Bars indicate 50 µm.** (C)** Representative Immunohistochemically stained images and quantification of CD31 to measure new vessels in carotid plaques. Bars indicate 50 µm.The data are shown as the mean ±SEM, n=6. *P<0.05 vs. NS; #*P*<0.05 vs. GFP. RCA, right carotid arteries. NS, normal saline mice; GFP, lentivirus-GFP mice; shDKK1, lentivirus DKK1 shRNA; DKK1, lentivirus DKK1.

The results of immunohistochemical analysis of DKK1 demonstrated that DKK1 protein expression was significantly lower in the shDKK1 group and higher in the DKK1 group than in the NS and GFP groups** (Figure 1A),** which established that the overexpression and silencing vectors were effective. In the immunohistochemical analysis, the intraplaque expression of VEGF-A, VEGFR-2, MMP-2 and MMP-9 was intense in the control group but was significantly downregulated with DKK1 knockdown, and upregulated with DKK1 overexpression **(Figure 1B)**. Immunohistochemical analysis of the intraplaque expression of CD31 further confirmed that DKK1 increased plaque-associated angiogenesis **(Figure 1C).**

### DKK1 participated in angiogenesis in ox-LDL-induced HUVECs via the CKAP4/PI3K pathway

**Figure 2:**
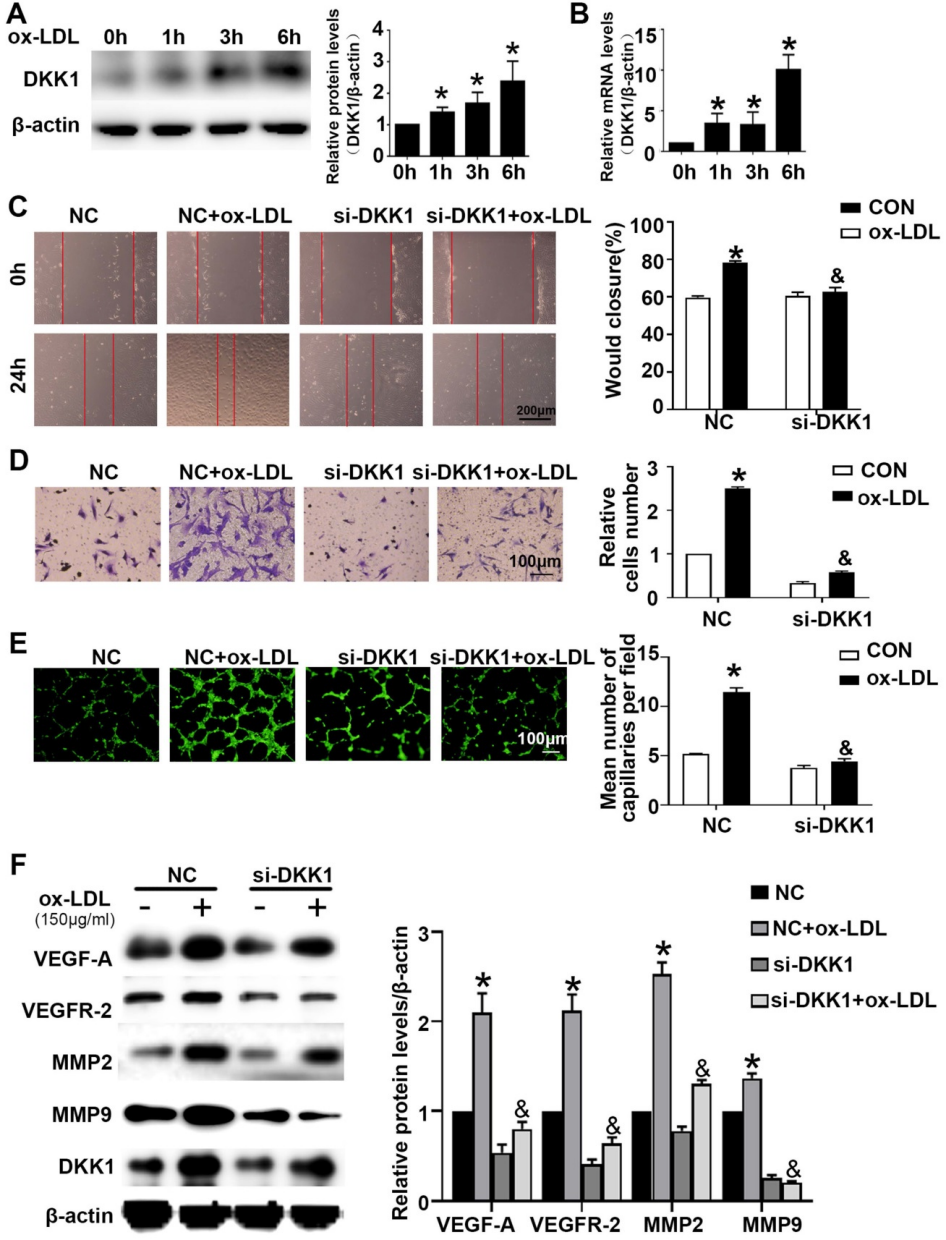
** Time-dependent effects of ox-LDL treatment on the expression of DKK1 and DKK1 participates in antiangiogenic effects in ox-LDL induced HUVECs. (A-B)** Quantification of the DKK1 level in HUVECs treated with ox-LDL (150 µg/ml) for various durations (0 h, 1 h, 3 h, 6 h): (A) DKK1 protein expression levels. (B) DKK1 mRNA levels. **(C-F)** HUVECs were transiently transfected with the negative control (NC) and DKK1 siRNA (si-DKK1) for 24 h and then treated with 150 µg/ml ox-LDL for 6 h. (C) Representative images and quantification of cell migration in the *in vitro* scratch wound assay (relative to 0 h) at 0h and 24 h post-wounding. Bars indicate 200 µm. (D) Representative images and quantification of cell migration in Transwell assays. The cell counts on the bottom of the Transwell are shown here. Bars indicate 100 µm. (E) Representative image and quantification of tube length (% of control). Bars indicate 100 µm. (F) Western blot to quantify VEGF-A, VEGF-2, MMP2 and MMP9 protein levels. The data are shown as the mean ±SEM. n=6. *P<0.05 vs. the untreated group or NC; &*P*<0.05 vs. NC+ ox-LDL.

HUVECs were treated with ox-LDL for different durations (0 h, 1 h, 3 h, 6 h). Western blotting and PCR showed that HUVECs treated with ox-LDL had increased DKK1 expression **(Figure 2A and 2B).** Compared with the NC+ox-LDL group, the si-DKK1+ox-LDL group showed less migration and tube formation **(Figure 2C-2E)**. siRNA transfection downregulated the expression of DKK1 and inhibited the ox-LDL-induced upregulation of VEGF-A, VEGFR-2, MMP-2 and MMP-9 **(Figure 2F)**. There was no difference between the NC+ox-LDL group and the si-DKK1+ox-LDL group in the EdU examination **(Figure S1A)**. The results indicated that DKK1 is involved in migration and angiogenesis but not proliferation in ox-LDL-treated HUVECs.

**Figure 3:**
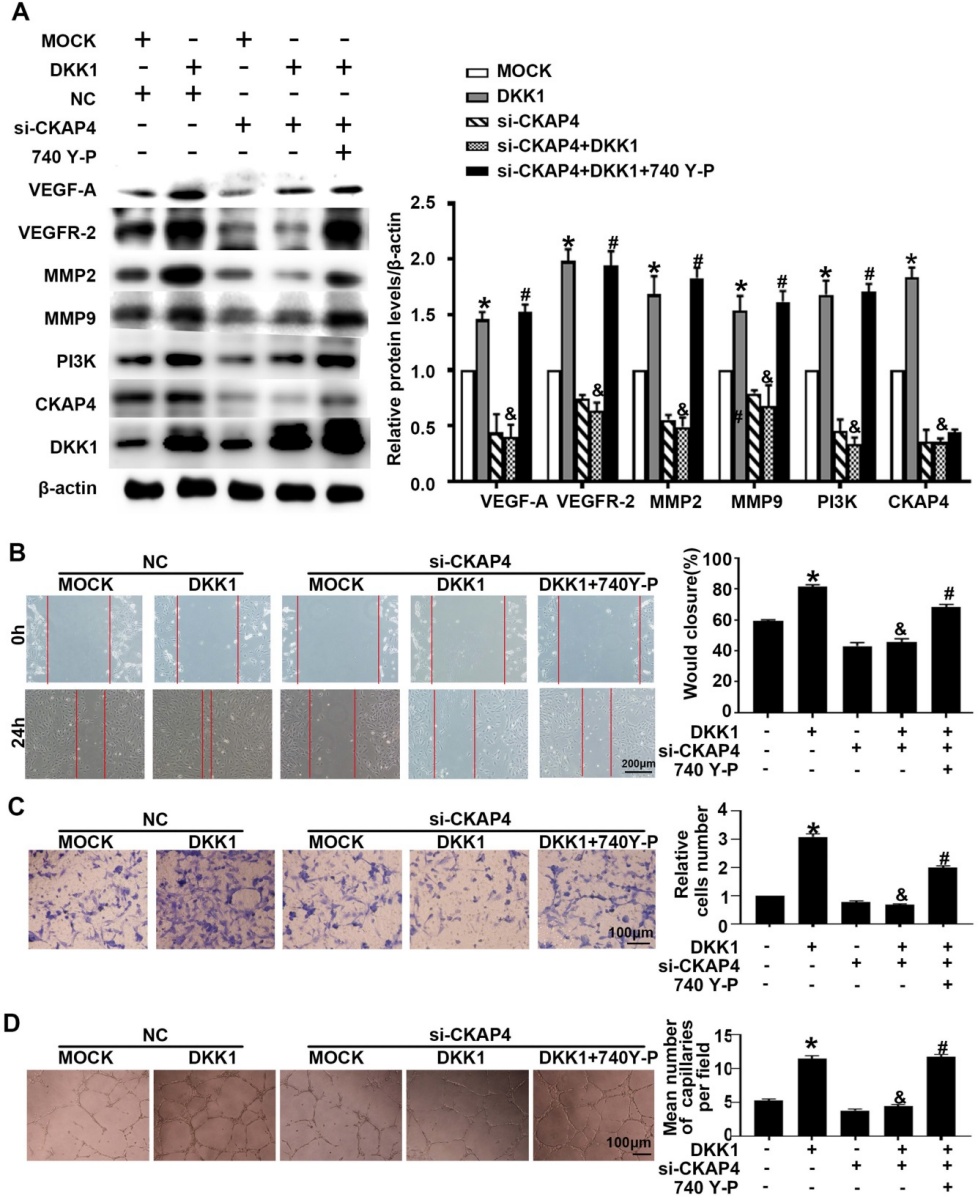
** DKK1 induces migration and angiogenesis by promoting CKAP4/PI3K.** Cells were pretreated with PBS or 740 Y-P (30 µM) for 12 h before transfection with lenti-DKK1 and DKK1 siRNA (si-DKK1) for 24 h.** (A)** Western blot for quantification of PI3K,VEGF-A,VEGF-2,MMP2 and MMP9 protein levels. **(B)** Representative images and quantification of cell migration in the *in vitro* scratch wound assay (relative to 0 h) from 0h and 24 h post-wounding. Bars indicate 200 µm. **(C)** Representative images and quantification of cell migration in Transwell assays. The cell counts on the bottom of the Transwell are shown here. Bars indicate 100 µm. **(D)** Representative image and quantification of the tube length (% of control). Bars indicate 100 µm. The data are shown as the mean ±SEM. n=6. *P<0.05 vs. MOCK; &*P*<0.05 vs. DKK1 transfection only; #*P*<0.05 vs. DKK1 and si-CKAP4 cotransfection.

IM-12 is an agonist of the canonical Wnt pathway. There was no difference in angiogenesis markers (VEGF-A, VEGFR-2, MMP-2 and MMP-9) between the DKK1 group and the DKK1+IM-12 group **(Figure S1B)**. In the DKK1 group, the expression of CKAP4 was upregulated **(Figure 3A)**. siRNA transfection downregulated the expression of CKAP4 and inhibited the ox-LDL-induced upregulation of angiogenesis markers (VEGF-A, VEGFR-2, MMP-2 and MMP-9), while 740 Y-P, a PI3K agonist, restored the upregulation **(Figure 3A)**. Compared with the DKK1 group, the DKK1+si-CKAP4 group showed less migration and tube formation, while 740 Y-P restored the migration and tube formation** (Figure 3B-3D)**. The results indicate that DKK1/CKAP4/PI3K is involved in angiogenesis in ox-LDL-treated HUVECs.

### Ets-1 participated in the ox-LDL-induced upregulation of DKK1 in HUVECs at the transcriptional level

**Figure 4:**
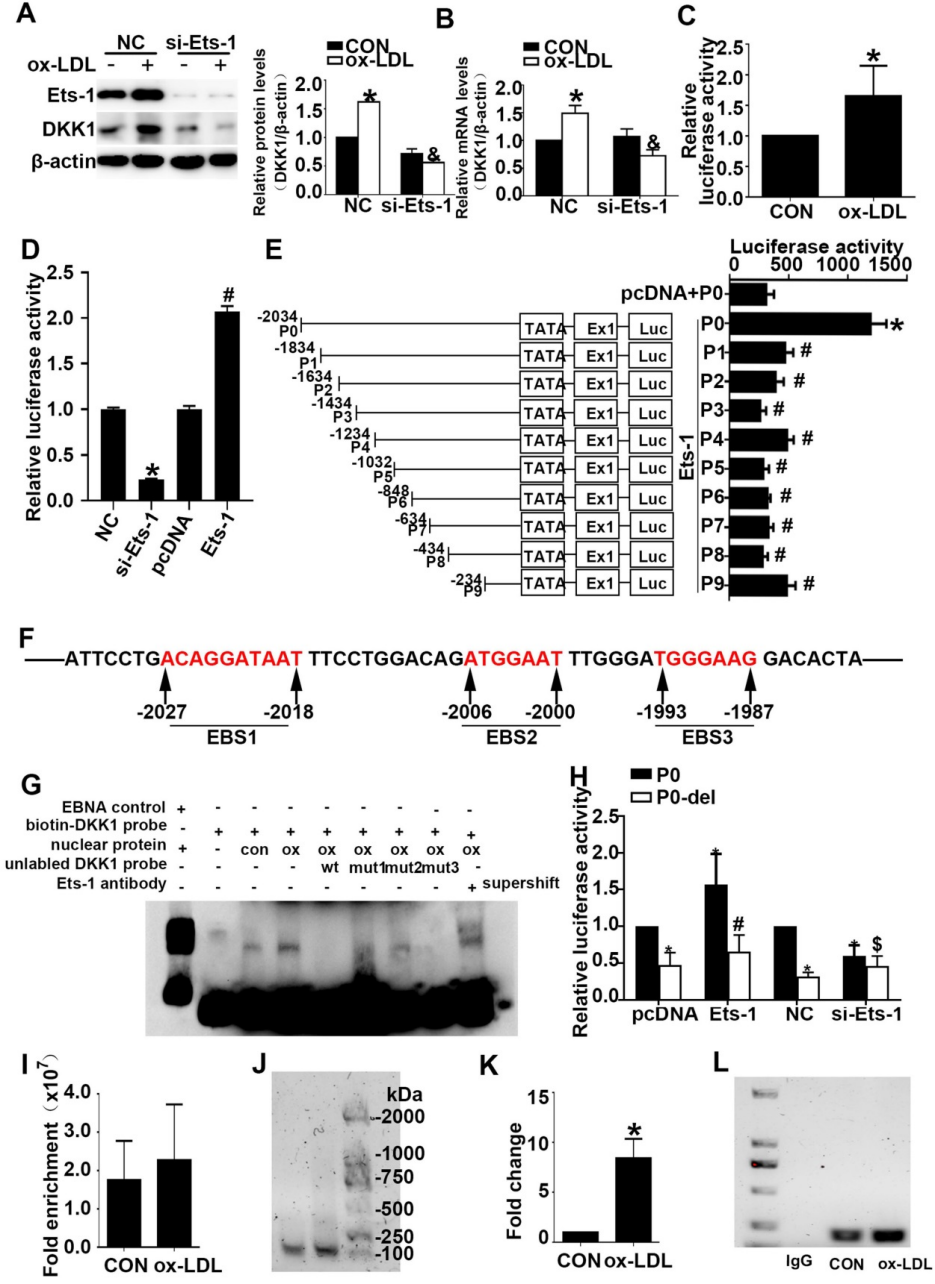
** Ets-1 promotes the expression of DKK1 by binding with a key positive regulatory region of the human DKK1 promoter. (A-B)** HUVECs were transiently transfected with the negative control (NC) and Ets-1 siRNA (si-Ets-1) for 24 h and then treated with 150 µg/ml ox-LDL for 6 h: (A) Western blot to quantify DKK1 protein levels; (B) qPCR to quantify DKK1 mRNA levels. **(C)** DLR assay in HUVECs transfected with the full-length DKK1 promoter and then treated with 150 µg/ml ox-LDL for 6 h. **(D)** DLR assay in 293T cells transfected with si-Ets-1 or PCDNA3.1-Ets-1 and the full-length DKK1 promoter. **(E)** DLR assay in 293T cells transfected with PCDNA3.1-Ets-1 and the larger serial fragment from -2034 to -234bp of the DKK1 promoter. **(F)** Prediction of Ets-1 binding sites in this region (-2034 ~ -1834bp) of the DKK1 promoter: EBS1 (-2023 to -2018bp), EBS2 (-2006 to -2000bp) and EBS3 (-1993 to -1987bp). **(G)** The oligonucleotides from -2036 to -1985bp of the DKK1 promoter were labeled with digoxin and incubated with nuclear extracts in the absence or presence of a 200-fold excess of unlabeled wild-type oligonucleotides or mutant oligonucleotides (mut1: mutant of the EBS1 element, mut2: mutant of the EBS2 element, and mut3: mutant of the EBS3 element). DNA-protein complexes were resolved by nondenaturing PAGE. **(H)** DLR assay in 293T cells transfected with si-Ets-1 (Ets-1 siRNA) or Ets-1 (PCDNA3.1-Ets-1 plasmids) and the deletion fragment promoter (-2006 ~ -2000bp) for 48 h. **(I-L)** The results of ChIP analysis show that Ets-1 was recruited to the DKK1 promoter regions in HUVECs. IgG was used as an immunoprecipitation control. The immunoprecipitated DNA was evaluated by real-time PCR and agarose gel electrophoresis. The data are presented as the mean ± SEM. n=6. *P<0.05 vs. the control group or NC; &*P*<0.05 vs. NC+ ox-LDL; #*P*<0.05 vs. P0+ Ets-1; $*P*<0.05 vs. P0+ si-Ets-1. PCDNA3.1 indicates basic plasmids, Ets-1 indicates PCDNA3.1-Ets-1 plasmids. NC indicates the negative control, si-Ets-1 indicates Ets-1 siRNA. CON indicates the untreated HUVEC group, ox-LDL indicates HUVECs treated with ox-LDL.

HUVECs were treated with ox-LDL for different durations (0 h, 1 h, 3 h, 6 h). Western blot and PCR showed that HUVECs treated with ox-LDL had higher Ets-1 expression **(Figure S2A and S2B)** than the 0 h group. Immunofluorescence showed that the nuclear translocation of Ets-1 increased after ox-LDL treatment **(Figure S2C)**. siRNA transfection downregulated the expression of Ets-1 and inhibited the ox-LDL-induced upregulation of DKK1** (Figure 4A and 4B)**. The results indicate that Ets-1 is involved in the regulation of DKK1 expression in ox-LDL-treated HUVECs.

Full-length, serially truncated and deletion fragment versions of the DKK1 promoter were cloned into the luciferase reporter vector pGL3-basic to generate pGL3- DKK1-promoter vectors, which were named P0, P1-P9 and P0-del, respectively. HUVECs were transiently cotransfected with the P0 and pRL-TK vectors and then exposed to ox-LDL for 6 h. A DLR assay showed that ox-LDL significantly increased the DKK1 promoter activity in HUVECs compared with the control. This finding indicated that ox-LDL could regulate DKK1 expression at the transcriptional level** (Figure 4C)**.

Cultured 293T cells were transiently cotransfected with the P0 and pRL-TK vectors and then transfected with PCDNA3.1-Ets-1, PCDNA3.1, negative control (NC) siRNA or Ets-1 siRNA. A DLR assay was performed and showed that Ets-1 siRNA decreased the DKK1 promoter activity in 293T cells** (Figure 4D)** and that Ets-1 significantly enhanced the promoter activity in 293T cells **(Figure 4D)**.

To identify a plausible regulatory promoter region, we generated a series of 5' deleted luciferase reporter constructs containing P (-2034, 1834, 1634, 1434, 1234, 1032, 848, 634, 434, 234)/luc fragments of the DKK1 promoter (groups P0 -P9). Cultured 293T cells were transiently cotransfected with the P0 vector, pRL-TK vector and PCDNA3.1 (control group) or cotransfected with the P0-P9 vectors, pRL-TK vector and PCDNA3.1-Ets1 (groups P0-P9). Compared to the control group, the P0 group showed a more than a 4-fold change, while the activity of the DKK1 promoter was significantly reduced from the P1 group to the P9 group **(Figure 4E)**.

To identify putative cis-acting elements and transcription factors contributing to DKK1 promoter activities in the region spanning -2034 to -1834 bp, the online prediction tools JASPAR and PROMO were used. Three possible loci, namely, EBS1, EBS2, and EBS3, existed at -2034 to -2018bp, -2006 to -2000bp, and -1993 to -1987bp, respectively **(Figure 4F)**. To explore these possible binding sites, we performed gel-shift assays using nuclear extracts from HUVECs. As shown in **Figure 4G**, a strong DNA complex was observed. The gel-shift experiment revealed that ox-LDL significantly increased DNA-protein complex formation. A 200-fold excess of wild-type cold competitive Ets-1 oligonucleotides (wt) eliminated the formation of the DNA/protein complex, while mutant cold competitive Ets-1 oligonucleotides (mut1, mut2, and mut3 for mutants of EBS1, EBS2, and EBS3, respectively) partially eliminated the formation of the DNA/protein complex** (Figure 4F)**. As shown in **Figure 4G**, the EBS3 mutant nearly abolished the formation of the DNA-protein complex, whereas the EBS1 and EBS2 mutants did not. Furthermore, EBS2 was least able to eliminate the formation of the DNA/protein complex** (Figure 4G, lane 7).** Therefore, the Ets-1 protein binds mainly to the EBS2 site of the DKK1 promoter. In the supershift lane, an obvious shift was found compared to the original position **(Figure 4G, lane 9)**. This finding indicates that Ets-1 is a potential transcription factor for the DKK1 gene. To further confirm the above result, cultured 293T cells were transiently cotransfected with the P0 vector (or P0-del), the pRL-TK vector and PCDNA3.1-Ets-1 (P0 group, P0-del group). Further deletion of 6 bp (from -2006 to -2000bp) resulted in a 50% decrease in promoter activity compared to that in the P0 group **(Figure 4H)**, suggesting an important role of EBS2.

As expected, the crosslinked DNA-Ets-1 complexes immunoprecipitated with an Ets-1 antibody were detected by PCR amplification with primers spanning the region of the DKK1 promoter from -2080 to -1894 bp. ChIP also revealed that, compared with the control, ox-LDL significantly increased the binding activity between the Ets-1 protein and the DKK1 promoter** (Figure 4I-4L)**. The results indicated that Ets-1 increased the transcriptional activity of the DKK1 promoter.

### ox-LDL induced Ets-1, CBP, and c-jun binding to DKK1 promoter in HUVECs

HUVECs were treated with ox-LDL for different durations. Western blot analysis showed that HUVECs treated with ox-LDL had higher c-jun and c-fos expression than the 0 h group **(Figure S2A and S2B)**. Immunofluorescence analysis showed that the nuclear translocation of c-jun, not c-fos, increased after ox-LDL treatment **(Figure S2C)**. siRNA transfection downregulated the expression of c-jun and inhibited the ox-LDL-induced upregulation of DKK1 **(Figure S2D and S2E)**. Cultured 293T cells were transiently cotransfected with the P0 and pRL-TK vectors and then transfected with NC siRNA or c-jun siRNA. A DLR assay showed that c-jun siRNA decreased DKK1 promoter activity in 293T cells** (Figure S2F)**. 293T cells were cotransfected with P0, pRL-TK and PCDNA3.1 (control group) or cotransfected with P0-P9, pRL-TK and PCDNA3.1-c-jun (group P0 -P9). A DLR assay showed that DKK1 promoter activity was increased in the P0 group and reduced from the P1 group to the P9 group **(Figure S2G)**. The online prediction tools JASPAR and PROMO were used to identify possible loci and binding sites (-2029 to -2023bp) **(Figure S2H)**. The results indicated that c-jun but not c-fos was involved in the regulation of DKK1 expression in ox-LDL-treated HUVECs.

**Figure 5:**
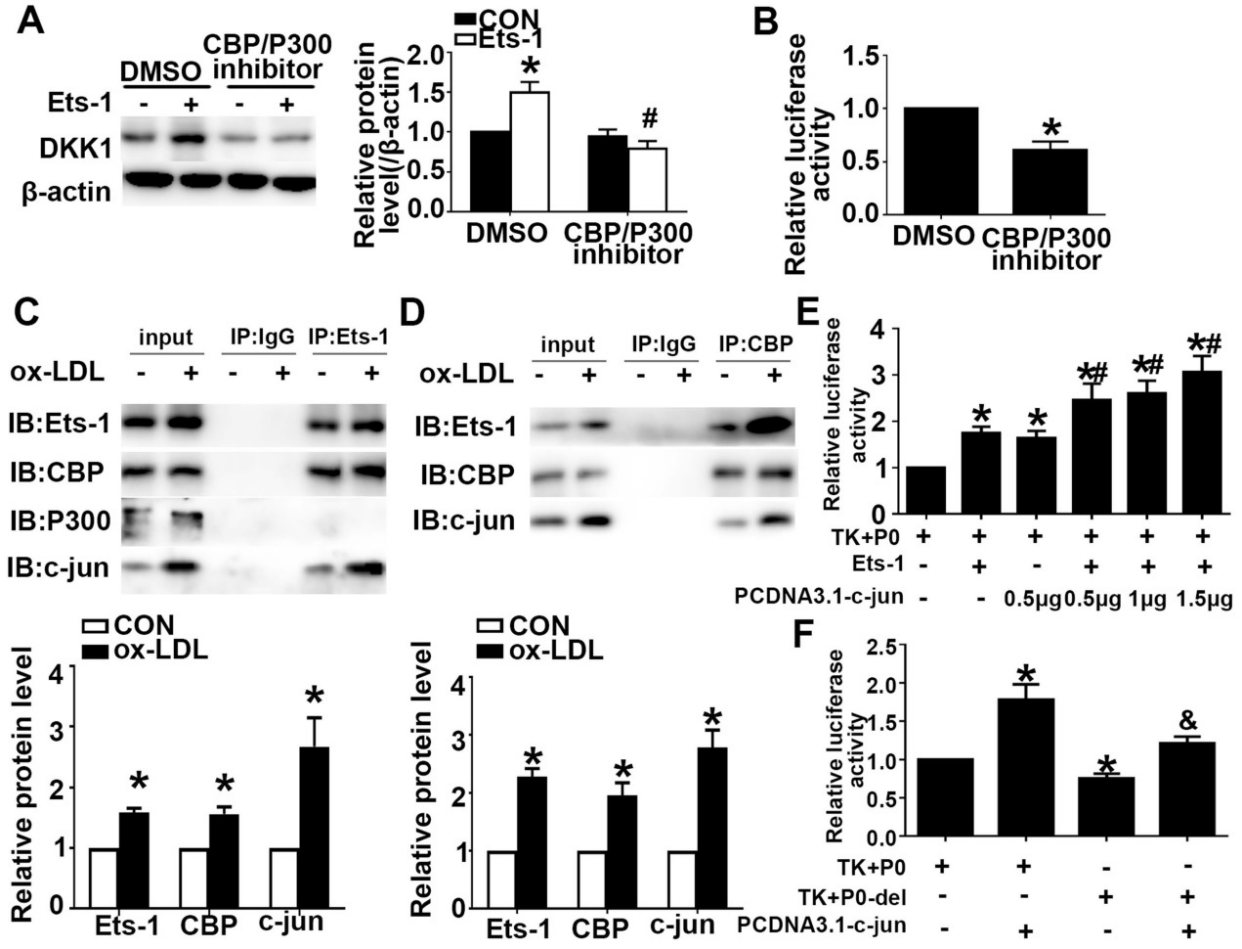
** CBP interacts with Ets-1 and assists in activating the expression of DKK1. (A)** HUVECs were pretreated with DMSO or a CBP/P300 inhibitor (25 µM) for 1 h after transfection with lenti-Ets-1. Western blot for quantification of the protein levels of DKK1. n=6. **(B)** HUVECs were pretreated with DMSO or CBP/P300 inhibitor (25 µM) for 1 h before transfection with the pGL3-full-length DKK1 promoter vector. The cells were collected, and the luciferase activities were analyzed. n=6. **(C)** Cell lysates were immunoprecipitated by an anti-Ets-1 monoclonal antibody, and the precipitates were then immunoblotted with an anti-CBP, anti-P300 or anti-c-jun antibody. n=3.** (D)** Cell lysates were immunoprecipitated by an anti-CBP monoclonal antibody, and the precipitates were then immunoblotted with anti-Ets-1 or anti-c-jun antibody. n=3.** (E)** 293T cells were cotransfected with PCDNA3.1-c-jun and PCDNA3.1-Ets-1. The luciferase activities were analyzed. n=6.** (F)** 293T cells were co-transfected with PCDNA3.1-c-jun and P0-del (or P0). The luciferase activities were analyzed. n=6. The data are presented as the mean ± SEM. *P<0.05 vs. the control group, #*P*<0.05 vs. the P0+Ets-1 group, &*P*<0.05 vs. the P0+c-jun group.

Previous studies have shown that Ets-1 can recruit the transcriptional coactivator CBP/P300 to target gene promoters and regulate gene expression. We showed that Ets-1 recruited the coactivator CBP to the DKK1 promoter. Pretreatment with a CBP/P300 inhibitor downregulated the expression of CBP/P300 and inhibited the ox-LDL-induced upregulation of DKK1 (**Figure 5A**). Cultured HUVECs were transiently cotransfected with the P0 and pRL-TK vectors and then treated with the CBP/P300 inhibitor for 12 h. A DLR assay was performed to determine the DKK1 promoter activity. The CBP/P300 inhibitor significantly decreased DKK1 promoter activity in 293T cells compared with that in the control group (**Figure 5B**). The results indicated that CBP but not P300 was involved in the regulation of DKK1 expression in ox-LDL-treated HUVECs. Coimmunoprecipitation (co-IP) was performed after treatment with ox-LDL. The results showed that Ets-1 interacted with the endogenous CBP and with c-jun but not with P300 (**Figure 5C**). With a reverse co-IP assay, we showed that CBP also interacted with endogenous c-jun and Ets-1 (**Figure 5D**). After cotransfection with PCDNA3.1-c-jun and PCDNA3.1-Ets-1, DKK1 promoter activity was increased compared with that for co-transfection with PCDNA3.1-Ets-1 alone (**Figure 5E**). After cotransfection with PCDNA3.1-c-jun and P0-del, the DKK1 promoter activity was decreased compared with that in the P0+ PCDNA3.1-c-jun group (**Figure 5F**). Taken together, these observations indicated that Ets-1, CBP and c-jun might form a complex to regulate DKK1 activity coordinately.

### Ets-1 was a functional target of miR-33a-5p and miR33a-5p eliminated angiogenesis in ox-LDL-induced HUVECs

**Figure 6:**
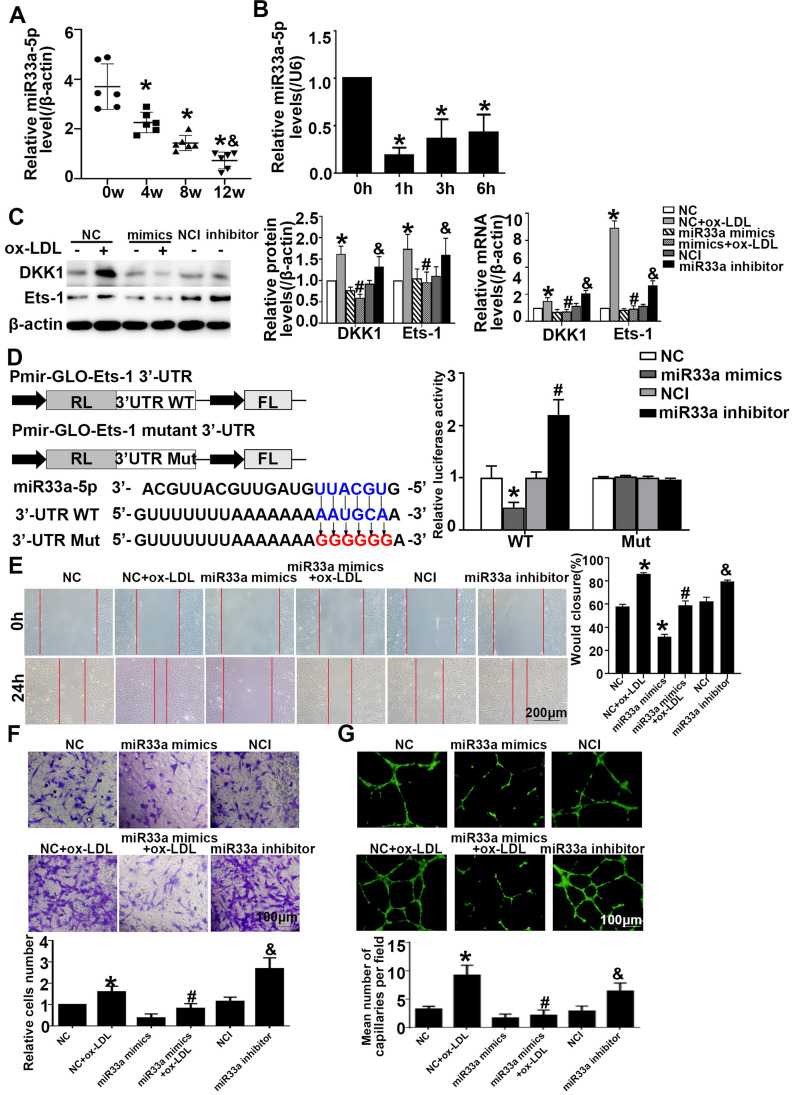
**miR33a-5p promotes the migration and angiogenesis of HUVECs by directly targeting and degrading Ets-1. (A)** miR33a-5p expression in the aortic artery in ApoE-/- mice treated with atherogenic chow for 0, 4, 8 and 12 weeks as determined by qRT-PCR. **(B)** PCR analysis of miR33a-5p in HUVECs treated with ox-LDL (150 µg/ml) for various lengths of time (0 h, 1 h, 3 h, and 6 h). **(C)** Western blot analysis of Ets-1 and DKK1 after miR33a-5p mimic or miR33a-5p inhibitor transfection in ox-LDL-treated HUVECs.** (D)** Possible binding sites for miR33a-5p in the Ets-1 3'-UTR, as predicted. A miR target reporter luciferase assay was performed after the miR33a-5p mimic and inhibitor were delivered to 293T cells. The results were normalized to data obtained from an assay with Renilla luciferase. **(E)** Representative images and quantification of cell migration in Transwell assays in miR33a-5p mimic-or miR33a-5p inhibitor-transfected HUVECs. The cell count on the bottom of the Transwell shown here. Bars indicate 200 µm. **(F)** Representative images and quantification of cell migration in the *in vitro* scratch wound assay (relative to 0h) from 0h and 24h post-wounding in miR33a-5p mimic or inhibitor-transfected HUVECs. Bars indicate 100 µm. **(G)** Representative image and quantification of the tube length (% of control) of the miR33a-5p mimic or inhibitor-transfected HUVECs. Bars indicate 100 µm. The data are presented as the mean ± SEM. n=6. *P<0.05 vs. the NC group; #*P*<0.05 vs. NC+ ox-LDL; &*P* <0.05 vs. NCI.

To examine potential miRNAs that negatively regulate DKK1 or Ets-1, a bioinformatics approach using multiple prediction algorithms (miRBase, PicTar, and TargetScan v6.1) was used to identify binding sites for miRNAs in the 3'-UTR of DKK1 and Ets-1. This analysis identified miR33a-5p as a potential regulator of DKK1 and Ets-1. ApoE^-/-^ mice were given atherogenic chow for 0, 4, 8 and 12 weeks, and miR33a-5p expression in the aortic artery was found to decrease with time (**Figure 6A**). HUVECs were treated with ox-LDL for different durations. qRT-PCR showed that ox-LDL downregulated the miR33a-5p expression in HUVECs compared with that in the 0 h group (**Figure 6B**). Dicer siRNA transfection upregulated the expression of DKK1 and Ets-1 (**Figure S3A**). Mimic transfection downregulated the expression of DKK1 and Ets-1, while inhibitor transfection upregulated the expression of DKK1 and Ets-1 (**Figure 6C**).

Transient cotransfection of miR33a-5p mimics with Ets-1 3'-UTR luciferase reporter plasmids resulted in significant repression of luciferase reporter gene expression in 293T cells, whereas cotransfection of 293T cells with NC miRNA or the mutants did not have any effect on luciferase expression. Transient cotransfection of the miR33a-5p inhibitor with Ets-1 3'-UTR luciferase reporter plasmids gave the opposite result (**Figure 6D**). The results indicated that miR33a-5p was involved in the regulation of Ets-1 and DKK1 and directly bound to the 3'-UTR of Ets-1. Transient cotransfection of miR33a-5p mimics with DKK1 3'-UTR luciferase reporter plasmids did not have any effect on the expression of luciferase in 293 cells, and cotransfection of 293T cells with NC miRNA or the mutants also did not have any effect on the expression of luciferase. Transient cotransfection of the miR33a-5p inhibitor with DKK1 3'-UTR luciferase reporter plasmids gave the same result (**Figure S3B**). The results indicated that miR33a-5p was involved in the regulation of Ets-1 and DKK1 and bound directly to the 3'-UTR of Ets-1.

Compared with the NC+ox-LDL group, the miR33a-5p mimics+ox-LDL group showed less migration and tube formation (**Figure 6E-6G**). Compared with the NCI group, the miR33a-5p inhibitor group showed more migration and tube formation (**Figure 6E-6G**). The results indicated that miR33a-5p eliminated migration and angiogenesis of ox-LDL-induced HUVECs.

### miR33a-5p exhibited antiangiogenic effect in ox-LDL-induced HUVECs via degrading Ets-1/DKK1

**Figure 7:**
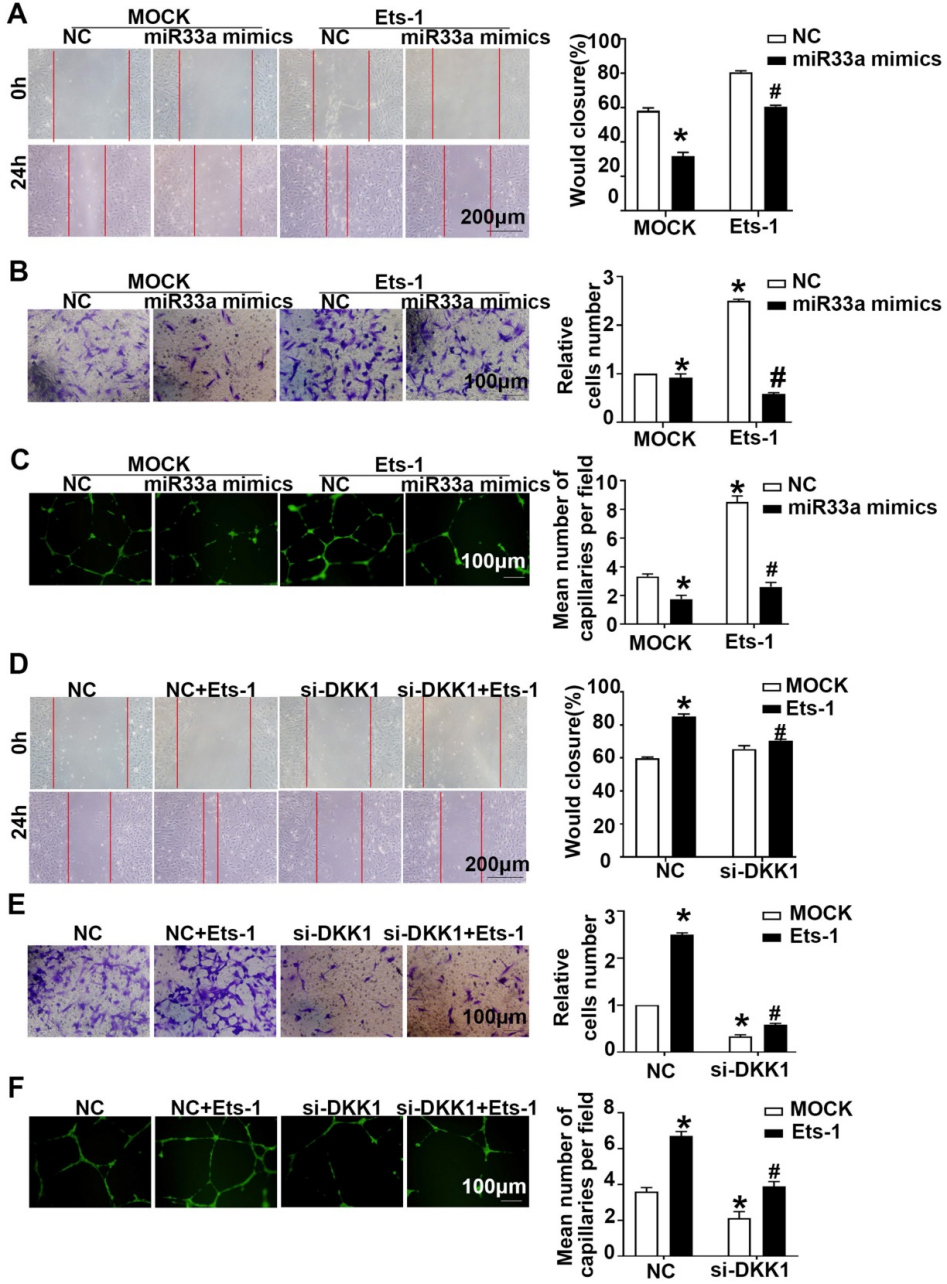
** miR33a-5p exhibits antiangiogenic effects in ox-LDL induced HUVECs by degrading Ets-1/DKK1. (A, D)** Representative images and quantification of cell migration in the *in vitro* scratch wound assay (relative to 0h) were obtained at 0h and 24h post-wounding, bars indicate 200 µm: (A) in miR33a mimic-transfected HUVECs before transfected lenti-Ets-1; (D) in DKK1 siRNA-transfected HUVECs after transfection with lenti-Ets-1. **(B, E)** Representative images and quantification of cell migration in Transwell assays, bars indicate 100 µm: (B) miR33a-5p mimic-transfected HUVECs before transfection with lenti-Ets-1; (E) DKK1 siRNA-transfected HUVECs after transfection with lenti-Ets-1. The cell counts on the bottom of the Transwell are shown here.** (C, F)** Representative image and quantification of the tube length (% of control), bars indicate 100 µm: (C) in the miR33a-5p mimic-transfected HUVECs before transfection with lenti-Ets-1; (F) in the DKK1 siRNA-transfected HUVECs after transfection with lenti-Ets-1. The data are presented as the mean ± SEM. n=6. *P<0.05 vs. the NC group; #*P*<0.05 vs. miR33a-5p mimic or Ets-1. NC, Negative control; Ets-1, lentivirus Ets-1.

Compared with the miR33a-5p mimics group, the miR33a-5p mimics+lenti-Ets-1 group showed elevated migration and tube formation (**Figure 7A-7C**). Compared with the miR33a-5p inhibitor group, the miR33a-5p inhibitor +Ets-1 siRNA group showed less migration and tube formation (**Figure S4A-S4C**). The results suggest that miR33a-5p leads to ox-LDL-induced migration and tube formation by inhibiting Ets-1 in ECs.

Compared with the NC group, the NC+lenti-Ets-1 group showed more migration and tube formation. Compared with the NC+lenti-Ets-1 group, the DKK1 siRNA+lenti-Ets-1 group showed less migration (**Figure 7D-AF**). These results suggest that Ets-1 causes the migration of ECs and tube formation by inducing DKK1.

Next, compared with the lenti-Ets-1 group, the CBP/P300 inhibitor+lenti-Ets-1 group showed less migration and tube formation (**Figure S4D-S4F**). This result suggests that CBP/P300 participates in the Ets-1-induced migration and tube formation.

## Discussion

In this study, we found that DKK1, a tumorigenesis associated molecule, promoted angiogenesis of carotid atherosclerotic plaques in high fat fed ApoE-/- mice. ox-LDL stimulates the secretion of TNF-α from macrophages and cytokines from ECs, indicates the starting point of atherosclerosis. To further explore the findings *in vivo*, ox-LDL was used to stimulate HUVECs *in vitro* in our study 21. We demonstrated that the high expression of DKK1 under ox-LDL stimulus increased the migration and angiogenesis in HUVECs via CKAP4/PI3K pathway. Data from Western blotting, real-time RT-PCR, electrophoretic mobility shift assay (EMSA) and ChIP revealed that upstream nuclear transcription factor Ets-1 could bind to the DKK1 promoter region, form a complex with CBP and c-jun, increase the transcriptional activity of the DKK1 promoter and promote DKK1 expression in the ox-LDL-treated HUVECs. Meanwhile, miR33a-5p was found to directly target the 3'-UTR of Ets-1 to regulate the expression of Ets-1 and DKK1. To the best of our knowledge, this is the first study to reveal the role and the underlying mechanisms of DKK1 in angiogenesis under the treatment of ox-LDL in HUVECs.

Angiogenesis, also named neovascularization, refers to the growth of new blood vessels that sprout from existing blood vessels. It's a complex process involved in differentiation, proliferation, migration and maturation of endothelial cells. Studies showed that angiogenesis intra atherosclerotic lesions plays vital roles in plaque growth and instability 22, and is of vital importance in plaque progression, plaque destabilization and thromboembolic events. Several angiogenesis-related genes, like VEGF, platelet-derived growth factor (PDGF) and tumor growth factor-β (TGF-β) in endothelial cells were demonstrated to be induced by atherosclerotic risk factors, such as oxidative stress, inflammatory factors and mechanical forces 23,24. The VEGF family which consists of five closely related members, namely VEGF-A, B, C, D and placental growth factor, played important roles in angiogenesis. The release of VEGF-A and activation of VEGFR-2, a receptor for VEGF-A contributes significantly to promote intra atherosclerotic angiogenesis 25,26. There were also studies showed that non-coding RNA regulated endothelial proliferation, migration and tube formation, and ultimately affected angiogenesis 27. Researchers have explored to target the intra-plaque angiogenesis through inhibition of vascular endothelial growth factor signaling, glycolytic flux and fatty acid oxidation 28-30. However, although anti-angiogenesis therapy has widely used in cancer treatment, studies on the pharmacological inhibition of this phenomenon in AS are still scare 31. In our study, we found that DKK1 was highly expressed and promoted the angiogenesis via increasing the expression of VEGF-A, VEGFR-2, MMP-2 and MMP-9 in the atherosclerotic plaques. CD31 is a marker of angiogenesis 32. *In vivo*, we detected the expression of CD31 to assess angiogenesis. The *in vitro* study in the ox-LDL-treated HUVECs also vertified these findings, which may provide a new potential intervention target for the anti-angiogenesis therapy in AS.

DKK1, a secretory glycoprotein of the DKK family, had been found to play vital roles both in cancers and AS. At present, DKK1 has been developed as a serological marker for the diagnosis and prognosis evaluation of several cancers, and also a new target for cancer treatment. In the registered clinical trials, we found DKK1 antibody DKN-01 had been entered into clinical phase I or phase II trial in advanced biliary tract cancer 7, advanced liver cancer, cholangiocarcinoma, gastric cancer and other tumors, and completed a promising study in gastric/gastroesophageal junction cance**r** 3,33**.** Another DKK1 antibody BHQ880 had completed the clinical phase II experiment for multiple myeloma in year 2020. Oncological treatment may increase the morbidity and mortality of cardiovascular diseases (CVDs) as a side effect 4. Our previous studies have revealed that DKK1 could promote endothelial apoptosis 5, destroy the tight junctions of endothelium 34, disturb the lipid metabolism 35 and lead to the development of AS. Anti-DKK1 neutralizing antibodies have shown promise and might be beneficial for the treatment of CVDs 7. Recent studies reported that angiogenesis was a link between atherosclerosis and tumorigenesis 36. Investigating the role of DKK1 in neovascularization will benefit both for the cancer and CVD treatment at the same time. Therefore, we further revealed the role and underlying mechanisms of DKK1 in AS from the perspective of angiogenesis in this study. Our findings showed that DKK1 promoted the migration and angiogenesis via up-regulating the angiogenesis-related molecules by activation of CKAP4/PI3K pathway, which indicated that anti-DKK1 therapy may prevent tumor and meanwhile reduce unstable plaques 37. Furthermore, we explored the upstream regulation mechanism of DKK1. Previous studies have revealed several mechanisms upstream of DKK1, including the aspects of histone modification, transcription, posttranscriptional modification and posttranslational modification (phosphorylation, glycosylation). It was reported that transcription factors (YAP, β-catenin, etc) binded to the DKK1 promoter region directly to activate its transcription 10,38,39. In this study, we found that nuclear transcription factors, c-jun and Ets-1, could bind to the DKK1 promoter region and activate DKK1 promoter. Using DLR, EMSA and ChIP experiments, we also clarified that Ets-1 binded to the -2006 to -2000bp region of the DKK1 promoter and c-jun binded to the -2029 to -2023bp region of the DKK1 promoter. Ets-1, a member of the Ets family of transcription factors, plays important roles in cellular proliferation, migration, vascular remodeling and apoptosis. Hypoxia, inflammatory factors and VEGF upregulate the expression of Ets-1 in ECs 14. Previous studies showed that Ets-1 performed multiple functions in ECs: (1) Ets-1 directly regulated several vascular genes, such as Flt1, Tek Kdr, Angpt2, Nrp1, Vwf, Pecam1 and Cdh5, to promote angiogenesis 13, and (2) Ets-1 directly upregulated MMPs and β3 integrin to promote migration 15,16. Feng, et al found that Ets-1 participated in inflammation induced carotid artery endoluminal vascular injury and promoted AS 40. We found that the expression of Ets-1 was significantly upregulated by ox-LDL in HUVECs, Ets-1 siRNA decreased DKK1 expression and relieved the ox-LDL-induced migration of ECs and angiogenesis, indicating that ox-LDL may cause changes through the Ets-1/DKK1 regulation of EC function. These findings further deepen our understanding of the regulatory mechanism of DKK1.

In addition, we also found that c-jun, one of AP-1 performs, upregulated the expression of DKK1 via increasing the promoter activity of it under the stimulus of ox-LDL. Previous studies have shown that c-jun promoted the expression of VEGF and induced angiogenesis 41. The transcription coactivators CBP and P300, which consist of four transcription factor binding domains (TADs) to recruit transcription factors, change chromatin superstructures, and activate acetylation 42, could promote the activation of a variety of transcription factors 43, such as c-jun (which binds to the CREB binding domain) 44, c-fos (which binds to third zinc finger domains) 45, and Ets-1 (which binds to first zinc finger domains) 46. The bindings of Ets-1, AP-1 14,47, and the coactivator CBP/P300 promote the connection of these two transcription factors 13. CBP/P300 is also involved in the acetylation of the DKK1 promoter in breast cancer 48. In our studies, we found protein binding among CBP, Ets-1 and c-jun. Ets-1 increased the transcriptional activity of DKK1 with the aid of c-jun and CBP. The effect of Ets-1 on migration and angiogenesis was reversed by a CBP/P300 inhibitor. The results indicated that CBP assisted in the effects of Ets-1/DKK1 on EC function.

MiRNAs are small (19-23 nucleotides) non-coding RNAs which regulate the target genes by binding to the 3' untranslated region (UTR) of mRNA post-transcriptionally. Studies have shown that miRNAs play vital roles in the development of AS and the pathological processes of ECs. Previous studies reported that the expression of Ets-1 in ECs can be inhibited by miR155 49, miR-199a-5p 13, the miR-200 family 50 and miR-221/222 49. In addition, miR-217 51, miR152 11, miR-376a 52 and other miRNAs decreased the expression of DKK1 by inhibiting translation or decreasing mRNA stability in different diseases. A previous study found that DKK1 is regulated by miR217, miR33a, miR33b, miR103a, miR93 and miR106a in diabetic cardiomyopathy 20. In this study, we further clarified the possible miRNAs in the regulation of Ets-1 and DKK1. Micro.org, STARBASE, TargetScan and other microRNA programs predicted that both Ets-1 and DKK1 had probable binding regions with the miR33a-5p (3'-UTR) binding sites. In a DLR assay, miR33a-5p bound to the Ets-1 3'-UTR but not the DKK1 3'-UTR, which suggested that miR33a-5p may inhibit the expression of Ets-1 and thus play a role in the regulation of DKK1. At present, there are few studies on miR33a. There are two subtypes of miR33 in humans, miR33a and miR33b 53. Human miR33a and the only mouse homologue (miR-33) are located at intron 16 of SREBP-2. It was reported to regulate the chemoresistance, proliferation, invasion and angiogenesis in tumor 54. MiR33a was also closely related to the lipid metabolism (inhibition of ABCA1 and cholesterol efflux, inhibition of HDL production) 18. Horie T, et al found that miR-33 deficiency reduced atherosclerotic plaque size and lipid content suggesting that miR-33 inhibition may prevent atherosclerosis progression 53. Talepoor, et al found that ECs exhibit a protective effect and inhibit miR-33a expression in monocytes, when cocultured with monocytes. The result indicated miR33a might play a protective role in the formation of atherosclerosis 55. As well, the expression of miR33a-5p decreased over time in the high fat fed ApoE-/- mice in our study, which indicating down-regulation of miR33a-5p in the development of atherosclerosis. However, whether miR33a affected plaque development by affecting endothelial cell function needs further proof. The effect of EC specific miR33a on atherosclerosis is the limitation of this study. In our study, we further explored whether miR-33a exerted great effects in the angiogenesis in the ox-LDL stimulated HUVECs and found that miR33a-5p directly bound to Ets-1 3'-UTR, decreased the expression of Ets-1 and DKK1, and attenuated the migration of endothelial cells and angiogenesis.

In conclusion, DKK1 promoted angiogenesis intra atherosclerotic plaques. DKK1 upregulated migration and angiogenesis of HUVECs via the CKAP4/PI3K pathway, not via Wnt/β-catenin pathway. miR-33a/Ets-1/DKK1 axis exerted important effects on the migration and angiogenesis in HUVECs under the stimulus of ox-LDL. The protein binding complex by CBP, c-jun and Ets-1 facilitated this process. Deep exploration of the underlying mechanisms in the angiogenesis in atherosclerosis is essential for the designing novel therapeutic targets, and is also important for the recognition of the cardiovascular side effects in the process of related anti-tumor treatment in future.

## Supplementary Material

Supplementary figures and tables.

## Abbreviations

AS: atherosclerosis
ChIP: chromatin immunoprecipitation
CKAP4: cytoskeleton-associated protein 4
co-IP: coimmunoprecipitation
CRDs: cysteine rich domains
CVDs: cardiovascular diseases
DKK1: Dickkopf1
EC: endothelial cell
EdU: Five-ethynyl-2'-deoxyuridine
EMSA: electrophoretic mobility shift assay
DLR: dual-luciferase reporter assay
HUVECs: human umbilical vein endothelial cells
IHC: Immunohistochemical analysis
miRNA: microRNA
NC: negative control
NS: normal saline
qRT-PCR: realtime quantitative polymerase chain reaction
RCA: right carotid artery
siRNA: small interfering RNA
3'-UTR: 3'-untranslated region
