# Supplementary material for: The pro-angiogenesis effect of miR33a-5p/Ets-1/DKK1 signaling in ox-LDL induced HUVECs
Source: Int J Biol Sci. 2021 Oct 3;17(15):4122–39. doi: 10.7150/ijbs.60302 (PMC8579465; doi:10.7150/ijbs.60302)
Supplement: Supplementary file 1 — Supplementary figures and tables. [file ijbsv17p4122s1.pdf]

SUPPLEMENTAL MATERIAL

The pro-angiogenesis effect of miR33a-5p/Ets-1/DKK1 signaling in ox-LDL induced HUVECs

Mingxue Di[1，2]; Yu Zhang[1]; Renya Zeng[1]; Xiaolin Liu[1]；Weijia Chen[1]; Meng Zhang[1]; Cheng Zhang[1]; Mengmeng Li[1]; Mei Zhang[1]

1 The Key Laboratory of Cardiovascular Remodeling and Function Research, Chinese Ministry of Education, Chinese National Health Commission and Chinese Academy of Medical Sciences, The State and Shandong Province Joint Key Laboratory of Translational Cardiovascular Medicine, Department of Cardiology, Qilu Hospital of Shandong University, Jinan, China

2 Department of Gerontology, The First Affiliated Hospital of Shandong First Medical University & Shandong Provincial Qianfoshan Hospital

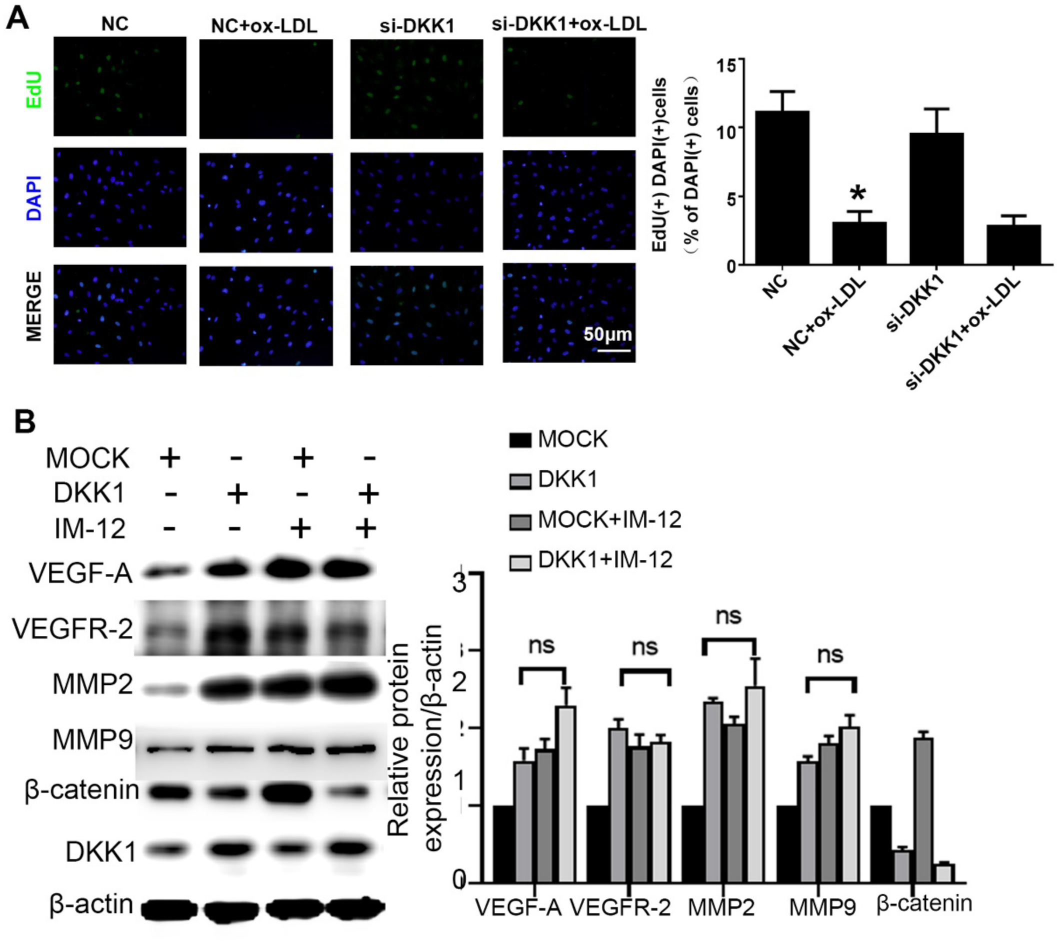

Figure S1. DKK1 did not participate in the proliferation of ox-LDL-treated HUVECs, and DKK1-induced angiogenesis did not occur via the canonical Wnt/β-catenin pathway. (A) HUVECs were transiently transfected with negative control (NC) and DKK1 siRNA (si-DKK1) for 24h and then treated with 150 μg/ml ox-LDL for 6h. The percentage of Edu (+) cells was calculated and quantified. Bars indicate 50 μm. **(B)** Cells were pretreated with DMSO or IM-12 (3 μM) for 1 h and then transfected with lenti-DKK1. Western blot to quantify the VEGF-A, VEGFR-2, MMP2, MMP9 and β-catenin protein levels. The data are shown as the mean±SEM. n=6. **P*<0.05 vs.NC.

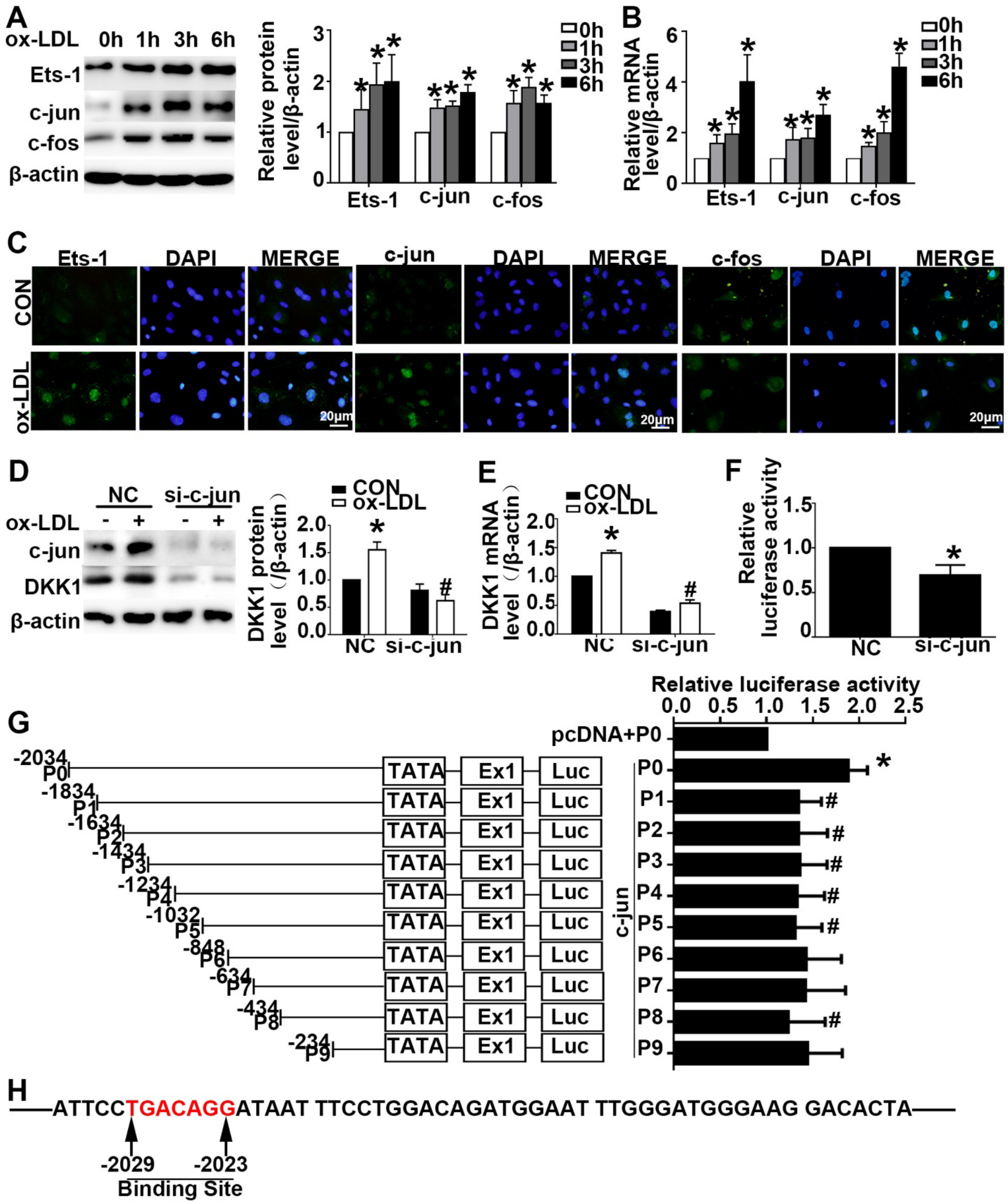

Figure S2. c-jun promotes the expression of DKK1 by binding with a key positive regulatory region of the human DKK1 promoter. (A-B) HUVECs treated with ox-LDL (150 μg/ml) for various lengths of time (0h, 1h, 3h, and 6h). Western blot and qRT-PCR to quantify the Ets-1, c-jun or c-fos protein and mRNA levels. **(C)** The immunofluorescence localization of Ets-1, c-jun or c-fos in HUVECs treated with ox-LDL (150 μg/ml) for 6h. Bars indicate 20 μm. **(D-E)** HUVECs were transiently transfected with NC or c-jun siRNA (si-c-jun) for 24h and then treated with 150 μg/ml ox-LDL for 6h. Western Blot and qRT-PCR were used to quantify the DKK1 protein and mRNA levels. **(F-G)** The luciferase activities were analyzed: **(F)** Full-length DKK1 promoter in 293T cells transfected with c-jun siRNA. **(G)** Rough characterization of the DKK1 promoter using a serial fragment from -2034 to -234bp. pcDNA3.0+P0, pcDNA3.0-c-jun +P0-P9 were transfected into 293T cells. **(H)** The prediction of c-jun binding sites in this region (-2034 ~ -1834bp) of the DKK1 promoter: Binding site (-2029 to -2023bp). The data are shown as the mean±SEM. n=6. **P*<0.05 vs. the untreated group or NC; #*P*<0.05 vs. NC+ox-LDL or P0+ PCDNA3.1-c-jun.

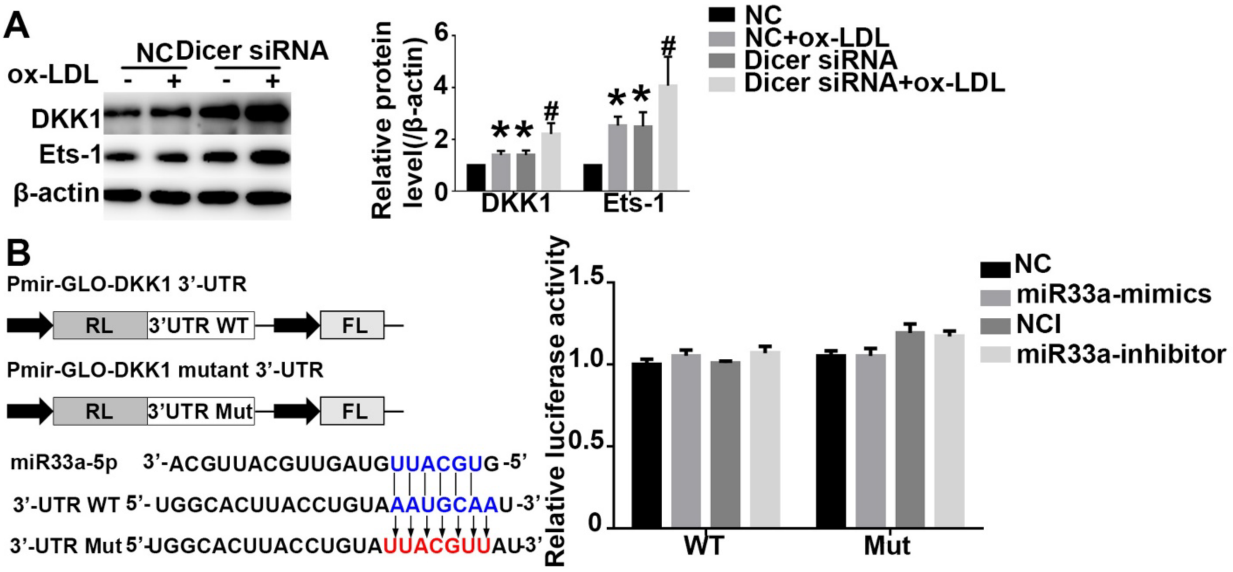

Figure S3. MiRNA participated in the upregulation of Ets-1 and DKK1 in ox-LDL-induced HUVECs, but miR33a-5p did not directly targeted DKK1. (A) HUVECs were transiently transfected with NC or Dicer siRNA for 24h and then treated with 150 µg/ml ox-LDL for 6h. Western blotting was used to quantify the Ets-1 and DKK1 protein levels. **(B)** Possible binding sites for miR33a-5p in the DKK1 3'-UTR, as predicted. A miR target reporter luciferase assay was performed after the miR33a-5p mimic and inhibitor were delivered to 293T cells. The results were normalized to data obtained from an assay with Renilla luciferase. The data are presented as the mean ± SEM. n=6. **P*<0.05 vs. the NC group or the control group; #*P*<0.05 vs. NC+ ox-LDL or P0+ PCDNA3.1-c-jun.

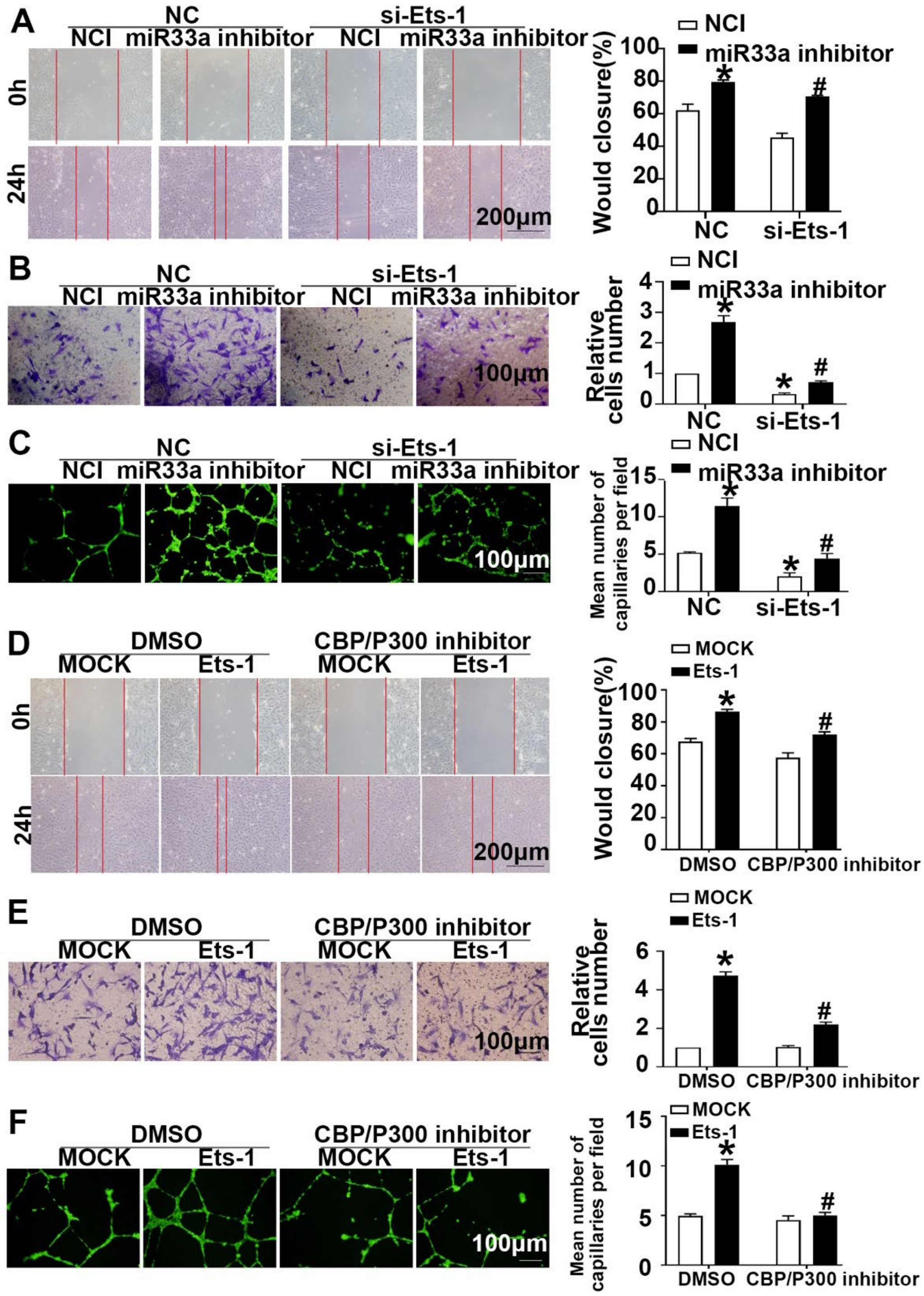

Figure S4. miR33a-5p/Ets-1 participated in the angiogenic effects in HUVECs via assistance of CBP/P300.

(A-C) HUVECs were cotransfected with an miR33a inhibitor and Ets-1 siRNA. **(D-F)** HUVECs were pretreated with DMSO or a CBP/P300 inhibitor (25 μM) for 1h after transfection with lenti-Ets-1(Ets-1). Representative images and quantification of cell migration in Transwell assays. The cell counts on the bottom of the Transwell are shown here: **(A, D)** Representative images and quantification of cell migration in Transwell assays. The cell counts on the bottom of the Transwell are shown here. Bars indicate 200 μm**. (B, E)** Representative images and quantification of cell migration in the in vitro scratch wound assay (relative to 0 h) were obtained at 0h and 24h post-wounding. Bars indicate 100 μm**. (C, F)** Representative image and quantification of the tube length (% of control). Bars indicate 100 μm**.** The data are presented as the mean±SEM. n=6. **P*<0.05 vs. the NCI group or the DMSO group; #*P*<0.05 vs. the miR33a-5p inhibitor or the DMSO+Ets-1 group.

Major Resources Table

Animals (in vivo studies)

Species	Vendor or Source	Background Strain	Sex
ApoE-/- mice	Beijing HFK Bioscience Co.,Ltd.	C57BL/6J	male

Antibodies

Target antigen	Vendor or Source	Catalog #	Working concentration
β-actin	Cell Signaling Technology	3700	WB(diluted1:1000)
DKK1	Abcam	ab109416	WB(diluted 1:500); IHC(diluted1:100)
P300	Abcam	ab14984	WB(diluted 1:500);
CBP	Abcam	ab2832	WB(diluted 1:500); IP(diluted1:50)
c-fos	Abcam	ab214672	WB(diluted 1:1000); IF(diluted1:200)
c-jun	Abcam	ab32137	WB(diluted 1:2000); IF(diluted1:200)
Ets-1	Abcam	ab96478(mice antibody)	WB(diluted 1:1000); IF(diluted1:400); ChIP (diluted 1:50)
Ets-1	Cell Signaling Technology	14069(rabbit antibody)	WB(diluted1:1000) ;IP (diluted 1:50)
IgG1	Cell Signaling Technology	3900	IP (diluted 1:50)
CD31	Abcam	ab281583	IHC(diluted1:50)
VEGF-A	Abcam	ab52917	WB(diluted 1:10000); IHC(diluted1:250)
VEGFR-2	Abcam	ab39638	WB(diluted 1:1000); IHC(diluted1:100)
MMP2	Abcam	ab37150	WB(diluted 1:1000); IHC(diluted1:50)
MMP9	Abcam	ab38898	WB(diluted 1:1000); IHC(diluted1:50)
PI3K	Cell Signaling Technology	4249	WB(diluted1:1000)
CKAP4	Abcam	ab152154	WB(diluted1:10000)
β-catenin	Cell Signaling Technology	8480	WB(diluted1:1000)

DNA/cDNA Clones

Clone Name	Sequence
β-actin primer	Forward: CGTGCGTGACATTAAGGAGA<br>Reverse: CACCTTCACCGTTCCAGTTT
DKK1 primer	Forward: ATAGCACCTTGGATGGGTATTCC<br>Reverse: CTGATGACCGGAGACAAACAG
Ets-1 primer	Forward:TACACAGGCAGTGGACCAATC<br>Reverse: CCCCGCTGTCTTGTGGATG
c-fos primer	Forward: CACTCCAAGCGGAGACAGAC<br>Reverse: AGGTCATCAGGGATCTTGCAG
c-jun primer	Forward: TCCAAGTGCCGAAAAAGGAAG<br>Reverse: CGAGTTCTGAGCTTTCAAGGT
U6 primer	Forward: CAGCACATATACTAAAATTGGAAGG<br>Reverse: ACGAATTTGCGTGTCATCC

miR33a-5p primer	Forward: CCTCATAAGCGGTGCATTGTA<br>Reverse:TATGCTTGTTCTCGTCTCTGTGTC
biotin-DKK1-S	TGACAGGATAATTTCCTGGACAGATGGAATTTGGGATGGGAAGGA
DKK1-S	TGACAGGATAATTTCCTGGACAGATGGAATTTGGGATGGGAAGGA
DKK1-A	TCCTTCCCATCCCAAATTCCATCTGTCCAGGAAATTATCCTGTCA
DKK1-MUT1-S	TGTAGCTGCACGTTCCTGGACAGATGGAATTTGGGATGGGAAGGA
DKK1-MUT1-A	TCCTTCCCATCCCAAATTCCATCTGTCCAGGAACGTGCAGCTACA
DKK1-MUT2-S	TGACAGGATAATTTCCTGGACAGGCTCGCATTGGGATGGGAAGGA
DKK1-MUT2-A	TCCTTCCCATCCCAATGCGAGCCTGTCCAGGAAATTATCCTGTCA
DKK1-MUT3-S	TGACAGGATGGTTTCCTGGACAGATGGAATTTGGGAGCTGCACGA
DKK1-MUT3-A	TCGTGCAGCTCCCAAATTCCATCTGTCCAGGAAATTATCCTGTCA
NC	Sense：UUCUCCGAACGUGUCACGUTT<br>Antisense：ACGUGACACGUUCGGAGAATT
c-fos siRNA	Sense: CUGUCAACGCGCAGGACUUTT<br>Antisense: AAGUCCUGCGCGUUGACAGTT
c-jun siRNA	Sense: CUGAUAAUCCAGUCCAGCATT<br>Antisense: UGCUGGACUGGAUUAUCAGTT
Ets-1 siRNA	Sense: AUGAUGUCUCAAGCAUUAAdTdT<br>Antisense: UUAAUGCUUGAGACAUCAUdTdT
CKAP4 siRNA	Sense:GGAAUGAUCUGGAUAGGUUTT<br>Antisense:AACCUAUCCAGAUCAUUCCTT
has-miR33a-5p-mimics	Sense: GUGCAUUGUAGUUGCAUUGCA<br>Antisense: CAAUGCAACUACAAUGCACUU
NCI	CAGUACUUUUGUGUAGUACAA
has- miR33a-5p-inhibitor	UGCAAUGCAACUACAAUGCAC

Cultured Cells and reagents

**Name**	**Source and working concentration**
HUVECs	ScienCell
HEK-293T	American Type Culture Collection
ox-LDL	Yiyuan Biotechnologies(Guangzhou, China), 150 μg/ml
CBP/P300 inhibitor	Abcam，ab142163,2ug/ml
IM-12	Selleck, S7566，3 μM
740 Y-P	Selleck, S7865, 50 μg/ml
